# Current Trends in Endoscopic Ear Surgery

**DOI:** 10.1097/ONO.0000000000000023

**Published:** 2022-12-02

**Authors:** Leona J. Tu, Manuela Fina, Justin S. Golub, Ken Kazahaya, Alicia M. Quesnel, Kareem O. Tawfik, Michael S. Cohen

**Affiliations:** 1 Drexel University College of Medicine, Philadelphia, PA; 2 Department of Otolaryngology—Head and Neck Surgery, University of Minnesota, Minneapolis, MN; 3 Department of Otolaryngology—Head and Neck Surgery, Valegos College of Physicians and Surgeons, NewYork-Presbyterian/Columbia University Irving Medical Center, New York, NY; 4 Division of Pediatric Otolaryngology, Children’s Hospital of Philadelphia, Philadelphia, PA; 5 Department of Otorhinolaryngology/Head and Neck Surgery, University of Pennsylvania, Philadelphia, PA; 6 Department of Otolaryngology—Head and Neck Surgery, Massachusetts Eye and Ear, Harvard Medical School, Boston, MA; 7 Department of Otolaryngology—Head and Neck Surgery, Vanderbilt University Medical Center, Nashville, TN.

Endoscopes have found broad applications within the field of otolaryngology by allowing direct visualization of hidden anatomic areas. However, while the endoscope has been robustly incorporated into standard practice in rhinology and laryngology for anterior skull base, sinus, and laryngeal surgery, its use in otology is emerging. Currently, the operating microscope is the preferred surgical instrument used by most otologists. Over the past 4 decades, however, use of the endoscope for otologic procedures has incrementally grown. In the 1990s, Tarabichi, Thomassin, and Poe utilized the endoscope for performing middle ear surgeries rather than purely for inspection, beginning a revolution in the way the endoscope is used in ear surgery (1–4). Many studies have since demonstrated the improved visualization of the tympanic cavity with the endoscope compared to the operating microscope, and clinical studies have shown outcomes of endoscopic ear surgery (EES) to be comparable to those of the operating microscope (5–13).

This article provides an overview of EES, including its history, current trends, advanced uses, integration into medical training, ergonomic and safety considerations, and utility of endoscopes in an office-based setting.

## HISTORY

Initially, the endoscope in otologic surgery was primarily used as an adjunct to the microscope and limited to diagnostic purposes. In the late 1980s to early 1990s, Nomura and Takahashi first reported transtympanic middle ear endoscopy (14,15). Poe and Bottrill used the technique to confirm perilymphatic fistula and to identify other middle ear pathologic conditions (16). Nearly a decade later, Thomassin used endoscopes to visualize residual cholesteatoma in the tympanic sinus and the retrotympanum (17). McKennan reported a second-look endoscopic inspection of mastoid cavities using a postauricular approach (18). In 1997, Tarabichi described EES for cholesteatoma without using a microscope (19). He subsequently described other procedures including endoscopic tympanoplasty and stapedotomy (2).

The works of Poe, Thomassin, and Tarabichi were pivotal in shaping the landscape of EES today (1–4). Technological advances in light sources and endoscopic cameras significantly improved the clarity of images. Endoscopic surgery, which previously required the surgeon to look through the endoscope with the naked eye, became more practical as video monitoring equipment improved, permitting quality visualization with preservation of the sterile field. In the 2000s, there was a growth in the use of transcanal endoscopic techniques to visualize hidden areas such as the posterior epitympanium (20). The International Working Group on Endoscopic Ear Surgery was created in 2007 to foster collaboration among otologists and to further the development of EES. Since then, EES has become increasingly used for all common otologic procedures (21–25). Clinical studies have shown outcomes following EES to be comparable to outcomes following microscopic ear surgery while allowing a less invasive approach (10–13,26).

## CURRENT TRENDS

A classification system introduced in 2018 defines EES and facilitates comparisons with traditional techniques (13). This classification ranges from class 0 (no endoscope) to class 3 (transcanal EES) and has helped resolve ambiguity in reporting outcomes with EES and enabled better communication among researchers (Table 1). A 2018 study found that the number of transcanal EES publications increased from 36 before 2010 to 283 between 2011 and 2018. This study also showed significant growth of Class 3 EES for cholesteatoma, from 14.9% in 2010 to 53.5% in 2018 among US surgeons. General endoscope use (regardless of EES class) for ear surgery also increased from 53.2% to 82.2%, highlighting the progressive movement towards incorporating the endoscope into mainstream practice in the United States (27).

**TABLE 1.** EES classification system

EES classification system					
EES Class	Class 0	Class 1	Class 2a	Class 2b	Class 3
Extent of endoscope usage	Microscope only (no endoscope)	Inspection only (endoscope used for inspection without dissection)	Mixed dissection (endoscope used for <50% of dissection)	Mixed dissection (endoscope used for >50% of dissection)	Endoscope only (no microscope)

Adapted from Cohen et al., 2018 (13).

EES indicates endoscopic ear surgery.

The expansion of EES has seen a similar trend around the world. The adoption of EES to manage middle-ear pathologies has been growing in North America, Asia, Europe, Africa, South America, and Australia (28–36). Recent surveys of otologists across the globe have demonstrated enthusiasm for incorporating EES into their practice (30,34,37).

## LATERAL SKULL BASE AND INNER EAR SURGERY

Advances in EES have prompted neurotologists to explore EES as a technique to manage lateral skull base and inner ear pathologies. Current management of these lesions primarily includes translabyrinth, transpetrosal, retrosigmoid, and middle cranial fossa approaches (38). However, these open, line-of-sight approaches may require wide craniotomy and prolonged brain retraction. Minimally invasive approaches have been explored using an endoscopic-assisted or exclusive endoscopic approach (39,40).

Three main endoscopic corridors for entering the lateral skull base have been described: the transcanal suprageniculate, transcanal transpromontorial, and transcanal infracochlear routes (38). These corridors can provide direct access for managing pathologies such as petrous apex cholesterol granulomas, vestibular schwannomas, intracochlear or intralabyrinthine schwannomas, and perilymph fistulas (41). The disadvantages of transcanal endoscopic lateral skull base surgery are similar to that of transcanal endoscopic middle ear surgery, only the risks are magnified. Simultaneous suction and dissection is challenging unless specialized combination instruments are available. At the same time, the relatively wider corridors may allow for endoscope holders and 2-handed surgery. Given the diversity in size and location of lateral skull base pathology, only certain types of tumors may be approachable through a transcanal corridor.

## MEDICAL EDUCATION

EES exposure varies significantly across training programs (42). The main challenge of EES is the difficulty of 1-handed dissection since the non-dominant hand must hold the endoscope. In 1 study of otolaryngologists and trainees, 44% reported single-handed surgery to be their main concern (30). Experienced surgeons who are accustomed to 2-handed dissection may find this transition particularly difficult (43).

One solution for overcoming these challenges is to increase early EES exposure by incorporating EES training into medical school and residency programs. In a study of resident perspectives of EES during surgical training, the majority of residents viewed EES favorably and expressed a desire to learn the technique (44). Medical students who were taught middle ear anatomy also perceived learning with endoscopes to be superior to microscopes (45,46). When surgical skill acquisition by students, residents, and attending physicians was compared using an endoscope or microscope, fewer injuries to the ossicular chain on cadavers were noted with endoscopes (47). A comparison of outcomes of endoscopic versus microscopic tympanoplasty during adoption of EES showed that similar outcomes in tympanic membrane closure, mean air-bone gap improvement, and mean duration of surgery can be maintained during the surgeon’s learning period while simultaneously teaching residents (48).

## ERGONOMICS

Operating for long periods of time in suboptimal postural positions can cause musculoskeletal disorders (MSDs) among surgeons. Otolaryngologists face an increased risk for MSDs (49,50). In surveys conducted in the United Kingdom, India, Canada, and Spain, 48%–97% of otolaryngologists reported work-related MSDs (49,51–53). Another survey of 325 otolaryngologists revealed that otologists suffered the highest pain among ENT subspecialties due to prolonged sitting and microscopic work (54). These findings highlight the importance of ergonomics when performing EES. Studies have shown that healthy posture improves operative performance and efficacy (55).

**FIG. 1.**
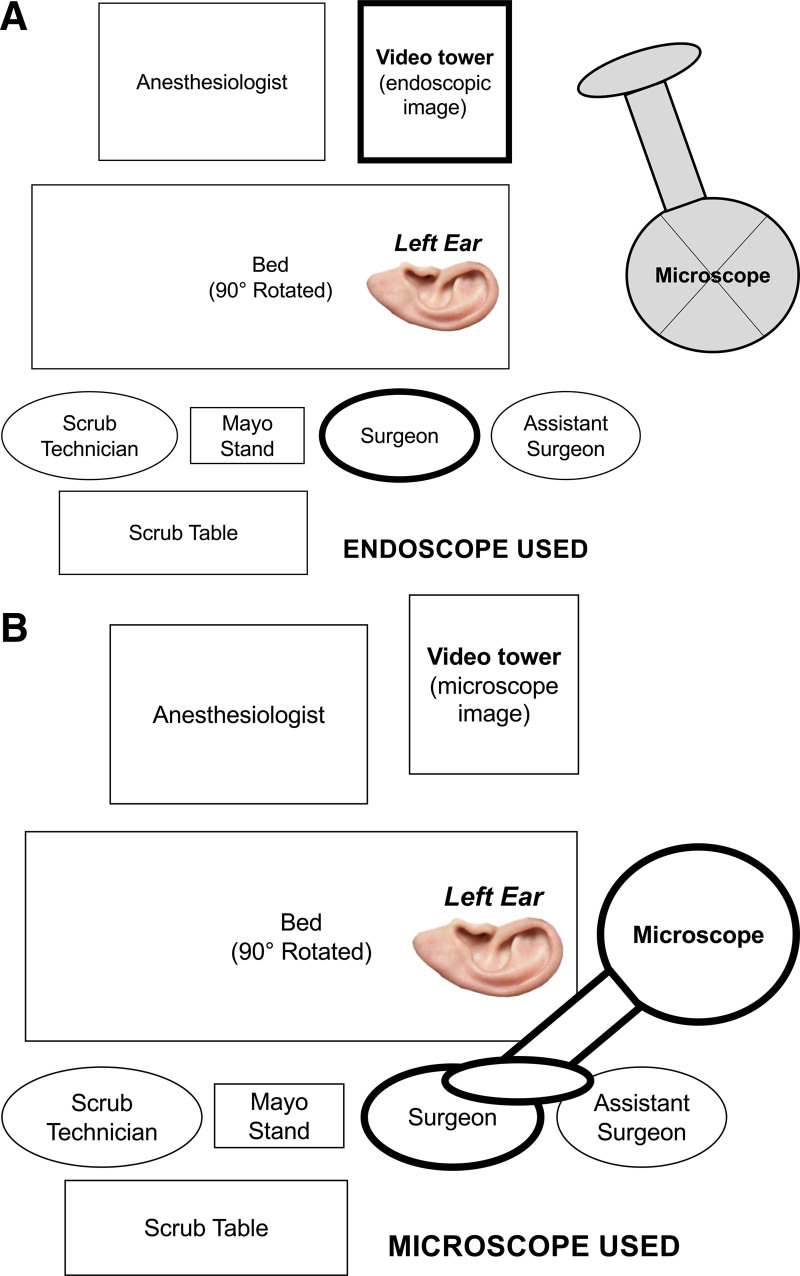
Comparison of operating room setups for endoscopic versus microscopic ear surgery. Ninety-degree rotation of the table with a left patient ear is depicted. *A,* Endoscope in use. The video tower is positioned directly in front of the surgeon. The surgeon, assistant surgeon, and scrub technician are sitting next to each other and facing a single screen, allowing for heads-up surgery. *B,* Microscope in use. The video tower is moved slightly away to accommodate the swinging microscope arm. The video tower is connected to the microscope and allows the scrub technician to view the image on the screen. However, the surgeon and assistant surgeon are required to operate by looking through the microscope oculars.

A challenge for otologists when working with microscopes is prolonged neck flexion. EES permits improved ergonomic posture by allowing for “heads up” surgery (Fig. 1). A neutral neck position can be achieved by placing the video tower directly in front of the surgeon with the monitor at eye level of the surgeon (41). Sitting in an upright position with elbows upon the bed or arm rests and utilizing back support can further maintain neutral neck, back, and arm positions (56).

## SAFETY

Numerous studies have documented the safety and efficacy of EES for treating common middle ear conditions such as cholesteatoma, otosclerosis, and tympanic membrane perforations (57,58). Nevertheless, there remain safety concerns when using endoscopes, primarily related to excessive heat (59). Studies have shown a positive correlation between endoscope diameter and heat, and elevated temperatures at the round window when using a xenon or LED light source, with the latter producing less heat (60,61). Desiccation of the chorda tympani nerve may be a potential risk (62,63).

It has also been reported that removing the endoscope from the ear can decrease the temperature within 20-88 seconds of turning off the light source and that application of suction or irrigation to the middle ear results in almost immediate temperature reduction (60,64). Thus, avoiding prolonged static positioning of the endoscope, decreasing the light intensity to the minimum effective level, and use of suction instrumentation or irrigation can improve EES safety (59,60,64,65).

## OFFICE-BASED OTOENDOSCOPY

The improved visualization provided by the endoscope applies to office-based otology procedures. Endoscopes allow for increased visualization of the depth of retraction pockets which can be difficult to assess with otomicroscopy (66). Endoscopic inspection of tympanic perforations can improve visualization of the anterior tympanic annulus and the entire margin of the perforation, which can be helpful for surgical planning. Preoperative endoscopic images can also be helpful for surgeons to review before surgery and to aid in patient education. Postoperatively, endoscopy can allow photo-documentation of outcomes and enable visualization to confirm healing after tympanoplasty for an anterior perforation. Innovative uses for in-office endoscopy include myringoplasty to repair small anterior perforations otherwise challenging to repair under microscopy and endoscopic debridement of mastoid cavities poorly accessible under microscopy (66).

## CONCLUSION

EES has seen gradual but increasingly significant developments over the past 4 decades. The rise in EES literature and changing attitudes have made it clear that EES is gaining interest among otologists around the world. Endoscopes are increasingly used for primary management of cholesteatoma and tympanic membrane perforations. Use of EES for treating other middle and inner ear conditions and lateral skull base disease has potential for continued growth.

## ACKNOWLEDGMENTS

None.

## FUNDING SOURCES

None declared.

## CONFLICT OF INTEREST

J.S.G. reported serving on the Alcon Surgical advisory board. K.K. is an Advanced Bionics Board Member and consultant for Cook Medical and serves on the Speaker Bureau for Cook Medical. K.K. also holds the position of Associate Editor for Otology and Neurotology Open and has been recused from reviewing and making decisions for the manuscript. A.M.Q. reported receiving sponsored research agreements from Grace Medical and Frequency Therapeutics, serving as a consultant to Alcon and Frequency Therapeutics, and owning a patent licensed to Grace Medical. K.O.T. served as an advisor to GlaxoSmithKline until October 2021. M.S.C. reported a sponsored research agreement with Med-El corporation. The remaining authors did not declare any conflicts of interest.

## DATA AVAILABILITY

Data sharing is not applicable to this article as no datasets were generated or analyzed during the current study.

## References

[1] Thomassin JM Inedjian JM Rud C Conciatori J Vilcoq P. Otoendoscopy: application in the middle ear surgery. Rev Laryngol Otol Rhinol (Bord). 1990;111:475–477. 2087611

[2] Tarabichi M. Endoscopic middle ear surgery. Ann Otol Rhinol Laryngol. 1999;108:39–46. 9930539 10.1177/000348949910800106

[3] Thomassin JM Korchia D Doris JM. Endoscopic-guided otosurgery in the prevention of residual cholesteatomas. Laryngoscope. 1993;103:939–943. 8361301 10.1288/00005537-199308000-00021

[4] Poe DS Rebeiz EE Pankratov MM Shapshay SM. Transtympanic endoscopy of the middle ear. Laryngoscope. 1992;102:993–996. 1518364 10.1288/00005537-199209000-00007

[5] Barakate M Bottrill I. Combined approach tympanoplasty for cholesteatoma: impact of middle-ear endoscopy. J Laryngol Otol. 2008;122:120–124. 17553186 10.1017/S0022215107009346

[6] El-Meselaty K Badr-El-Dine M Mandour M Mourad M Darweesh R. Endoscope affects decision making in cholesteatoma surgery. Otolaryngol Head Neck Surg. 2003;129:490–496. 14595271 10.1016/S0194-59980301577-8

[7] Kozin ED Gulati S Kaplan AB. Systematic review of outcomes following observational and operative endoscopic middle ear surgery. Laryngoscope. 2015;125:1205–1214. 25418475 10.1002/lary.25048 PMC4467784

[8] Tarabichi M. Endoscopic management of cholesteatoma: long-term results. Otolaryngol Head Neck Surg. 2000;122:874–881. 10828802 10.1016/S0194-59980070017-9

[9] Marchioni D Alicandri-Ciufelli M Molteni G Villari D Monzani D Presutti L. Ossicular chain preservation after exclusive endoscopic transcanal tympanoplasty: preliminary experience. Otol Neurotol. 2011;32:626–631. 21451429 10.1097/MAO.0b013e3182171007

[10] Presutti L Gioacchini FM Alicandri-Ciufelli M Villari D Marchioni D. Results of endoscopic middle ear surgery for cholesteatoma treatment: a systematic review. Acta Otorhinolaryngol Ital. 2014;34:153–157. 24882923 PMC4035841

[11] Choi N Noh Y Park W. Comparison of endoscopic tympanoplasty to microscopic tympanoplasty. Clin Exp Otorhinolaryngol. 2017;10:44–49. 27334511 10.21053/ceo.2016.00080 PMC5327595

[12] Hsu YC Kuo CL Huang TC. A retrospective comparative study of endoscopic and microscopic Tympanoplasty. J Otolaryngol Head Neck Surg. 2018;47:44. 29973286 10.1186/s40463-018-0289-4 PMC6033204

[13] Cohen MS Basonbul RA Barber SR Kozin ED Rivas AC Lee DJ. Development and validation of an endoscopic ear surgery classification system. Laryngoscope. 2018;128:967–970. 28782289 10.1002/lary.26802

[14] Nomura Y. Effective photography in otolaryngology-head and neck surgery: endoscopic photography of the middle ear. Otolaryngol Head Neck Surg. 1982;90:395–398. 6817266 10.1177/019459988209000406

[15] Takahashi H Honjo I Fujita A Kurata K. Transtympanic endoscopic findings in patients with otitis media with effusion. Arch Otolaryngol Head Neck Surg. 1990;116:1186–1189. 2206504 10.1001/archotol.1990.01870100080017

[16] Poe DS Bottrill ID. Comparison of endoscopic and surgical explorations for perilymphatic fistulas. Am J Otol. 1994;15:735–738. 8572084

[17] Thomassin JM. Otoendoscopically Guided Surgery. Berlin, Heidelberg: Springer; 1994.

[18] McKennan KX. Endoscopic “second look” mastoidoscopy to rule out residual epitympanic/mastoid cholesteatoma. Laryngoscope. 1993;103:810–814. 8341108 10.1288/00005537-199307000-00016

[19] Tarabichi M. Endoscopic management of acquired cholesteatoma. Am J Otol. 1997;18:544–549. 9303149

[20] Nogueira JF de Sousa Lobo Ferreira Querido R Goncalves da Silva Leite J Cabral da Costa T. Future of endoscopic ear surgery. Otolaryngol Clin North Am. 2021;54:221–231. 33153734 10.1016/j.otc.2020.09.023

[21] Badr-el-Dine M. Value of ear endoscopy in cholesteatoma surgery. Otol Neurotol. 2002;23:631–635. 12218610 10.1097/00129492-200209000-00004

[22] Cohen MS Landegger LD Kozin ED Lee DJ. Pediatric endoscopic ear surgery in clinical practice: lessons learned and early outcomes. Laryngoscope. 2016;126:732–738. 26228434 10.1002/lary.25410

[23] Migirov L Shapira Y Horowitz Z Wolf M. Exclusive endoscopic ear surgery for acquired cholesteatoma: preliminary results. Otol Neurotol. 2011;32:433–436. 21206389 10.1097/MAO.0b013e3182096b39

[24] Ayache S. Cartilaginous myringoplasty: the endoscopic transcanal procedure. Eur Arch Otorhinolaryngol. 2013;270:853–860. 22639200 10.1007/s00405-012-2056-x

[25] Tarabichi M. Endoscopic management of limited attic cholesteatoma. Laryngoscope. 2004;114:1157–1162. 15235340 10.1097/00005537-200407000-00005

[26] Kiringoda R Kozin ED Lee DJ. Outcomes in endoscopic ear surgery. Otolaryngol Clin North Am. 2016;49:1271–1290. 27565392 10.1016/j.otc.2016.05.008

[27] Kapadiya M Tarabichi M. An overview of endoscopic ear surgery in 2018. Laryngoscope Investig Otolaryngol. 2019;4:365–373. 10.1002/lio2.276 PMC6580051 31236473

[28] Kanona H Virk JS Owa A. Endoscopic ear surgery: a case series and first United Kingdom experience. World J Clin Cases. 2015;3:310–317. 25789304 10.12998/wjcc.v3.i3.310 PMC4360503

[29] Ito T Kubota T Furukawa T Matsui H Futai K Kakehata S. Transcanal endoscopic ear surgery for congenital middle ear anomalies. Otol Neurotol. 2019;40:1299–1305. 31634283 10.1097/MAO.0000000000002393

[30] Yong M Mijovic T Lea J. Endoscopic ear surgery in Canada: a cross-sectional study. J Otolaryngol Head Neck Surg. 2016;45:4. 26786729 10.1186/s40463-016-0117-7 PMC4717547

[31] Cohen MS Basonbul RA Kozin ED Lee DJ. Residual cholesteatoma during second-look procedures following primary pediatric endoscopic ear surgery. Otolaryngol Head Neck Surg. 2017;157:1034–1040. 28871887 10.1177/0194599817729136

[32] Emre IE Cingi C Bayar Muluk N Nogueira JF. Endoscopic ear surgery. J Otol. 2020;15:27–32. 32110237 10.1016/j.joto.2019.11.004 PMC7033590

[33] Sarkar S Banerjee S Chakravarty S Singh R Sikder B Bera SP. Endoscopic stapes surgery: our experience in thirty two patients. Clin Otolaryngol. 2013;38:157–160. 23164290 10.1111/coa.12051

[34] Ma AK Saxby A Kong J Patel N Jufas N. Endoscopic ear surgery in Australia. Aust J Otolaryngol. 2021;4:6–6.

[35] Preyer S. Endoscopic ear surgery - a complement to microscopic ear surgery. HNO. 2017;65:29–34. 27933350 10.1007/s00106-016-0268-x PMC5281654

[36] Bae MR Kang WS Chung JW. Comparison of the clinical results of attic cholesteatoma treatment: endoscopic versus microscopic ear surgery. Clin Exp Otorhinolaryngol. 2019;12:156–162. 30165729 10.21053/ceo.2018.00507 PMC6453796

[37] Agha-Mir-Salim P Kropp M Muller A. Endoscopic ear surgery in Germany: survey on the current situation and an international comparison. HNO. 2021;69:779–790. 34417641 10.1007/s00106-021-01094-1 PMC8378297

[38] Marchioni D Alicandri-Ciufelli M Rubini A Presutti L. Endoscopic transcanal corridors to the lateral skull base: initial experiences. Laryngoscope. 2015;125:S1–13. 10.1002/lary.25203 25703066

[39] Presutti L Nogueira JF Alicandri-Ciufelli M Marchioni D. Beyond the middle ear: endoscopic surgical anatomy and approaches to inner ear and lateral skull base. Otolaryngol Clin North Am. 2013;46:189–200. 23566905 10.1016/j.otc.2012.12.001

[40] Marchioni D Alicandri-Ciufelli M Rubini A Masotto B Pavesi G Presutti L. Exclusive endoscopic transcanal transpromontorial approach: a new perspective for internal auditory canal vestibular schwannoma treatment. J Neurosurg. 2017;126:98–105. 26967786 10.3171/2015.11.JNS15952

[41] Ridge SE Shetty KR Lee DJ. Current trends and applications in endoscopy for otology and neurotology. World J Otorhinolaryngol Head Neck Surg. 2021;7:101–108. 33997719 10.1016/j.wjorl.2020.09.003 PMC8103526

[42] Barber SR Chari DA Quesnel AM. Teaching endoscopic ear surgery. Otolaryngol Clin North Am. 2021;54:65–74. 33243377 10.1016/j.otc.2020.09.005

[43] Lucidi D Fernandez IJ Botti C. Does microscopic experience influence learning curve in endoscopic ear surgery? A multicentric study. Auris Nasus Larynx. 2021;48:50–56. 32680599 10.1016/j.anl.2020.06.015

[44] Ng CL Ong W Ngo RYS. Otolaryngology residents’ perceptions of endoscopic ear surgery during surgical training. J Laryngol Otol. 2020;134:233–240. 32114991 10.1017/S0022215120000365

[45] Anschuetz L Siggemann T Dur C Dreifuss C Caversaccio M Huwendiek S. Teaching middle ear anatomy and basic ear surgery skills: a qualitative study comparing endoscopic and microscopic techniques. Otolaryngol Head Neck Surg. 2021;165:174–181. 33287674 10.1177/0194599820977191

[46] Chern A Sharma RK Maurrasse SE Drusin MA Ciarleglio AJ Golub JS. Educational value of endoscopic versus microscopic ear surgery. Ann Otol Rhinol Laryngol. 2021;131:147–153. 10.1177/00034894211012600 33957787

[47] Anschuetz L Stricker D Yacoub A Wimmer W Caversaccio M Huwendiek S. Acquisition of basic ear surgery skills: a randomized comparison between endoscopic and microscopic techniques. BMC Med Educ. 2019;19:357. 31521153 10.1186/s12909-019-1803-8 PMC6744647

[48] Li B Asche S Yang R Yueh B Fina M. Outcomes of adopting endoscopic tympanoplasty in an academic teaching hospital. Ann Otol Rhinol Laryngol. 2019;128:548–555. 30793624 10.1177/0003489419830424

[49] Vijendren A Devereux G Kenway B. Effects of prolonged microscopic work on neck and back strain amongst male ENT clinicians and the benefits of a prototype postural support chair. Int J Occup Saf Ergon. 2019;25:402–411. 28965475 10.1080/10803548.2017.1386411

[50] Dahmash AB Alkholaiwi F Alahmari A Shadid AM Alharbi AM Al Hussain O. Work-related musculoskeletal symptoms in otorhinolaryngology-head and neck surgery residents. Sultan Qaboos Univ Med J. 2020;20:e202–e208. 32655913 10.18295/squmj.2020.20.02.011 PMC7328838

[51] Dabholkar T Yardi S Dabholkar YG Velankar HK Ghuge G. A survey of work-related musculoskeletal disorders among otolaryngologists. Indian J Otolaryngol Head Neck Surg. 2017;69:230–238. 28607896 10.1007/s12070-017-1106-5 PMC5446348

[52] Bolduc-Begin J Prince F Christopoulos A Ayad T. Work-related musculoskeletal symptoms amongst otolaryngologists and head and neck surgeons in Canada. Eur Arch Otorhinolaryngol. 2018;275:261–267. 29075981 10.1007/s00405-017-4787-1

[53] Lobo D Gandarillas MA Sanchez-Gomez S Megia R. Work-related musculoskeletal symptoms in otorhinolaryngology and their relationship with physical activity. A nationwide survey. J Laryngol Otol. 2019;133:713–718. 31317837 10.1017/S0022215119001452

[54] Babar-Craig H Banfield G Knight J. Prevalence of back and neck pain amongst ENT consultants: national survey. J Laryngol Otol. 2003;117:979–982. 14738610 10.1258/002221503322683885

[55] Ramakrishnan VR Montero PN. Ergonomic considerations in endoscopic sinus surgery: lessons learned from laparoscopic surgeons. Am J Rhinol Allergy. 2013;27:245–250. 23710962 10.2500/ajra.2013.27.3872

[56] Stern Shavit S Golub JS Lustig LR. The risks of being otologist, an ergonomic and occupational hazard review. Otol Neurotol. 2020;41:1182–1189. 32925834 10.1097/MAO.0000000000002769

[57] Kobayashi T Gyo K Komori M Hyodo M. Efficacy and safety of transcanal endoscopic ear surgery for congenital cholesteatomas: a preliminary report. Otol Neurotol. 2015;36:1644–1650. 26485586 10.1097/MAO.0000000000000857

[58] Bianconi L Gazzini L Laura E De Rossi S Conti A Marchioni D. Endoscopic stapedotomy: safety and audiological results in 150 patients. Eur Arch Otorhinolaryngol. 2020;277:85–92. 31624863 10.1007/s00405-019-05688-y

[59] Tarabichi M. Endoscopic transcanal middle ear surgery. Indian J Otolaryngol Head Neck Surg. 2010;62:6–24. 23120674 10.1007/s12070-010-0007-7 PMC3450149

[60] Kozin ED Lehmann A Carter M. Thermal effects of endoscopy in a human temporal bone model: implications for endoscopic ear surgery. Laryngoscope. 2014;124:E332–E339. 24604692 10.1002/lary.24666 PMC4465246

[61] Das A Mitra S Agarwal P Sengupta A. Prolonged intra-operative thermal exposure in endoscopic ear surgery: is it really safe? J Laryngol Otol. 2020;134:727–731. 32830635 10.1017/S0022215120001449

[62] McManus LJ Stringer MD Dawes PJ. Iatrogenic injury of the chorda tympani: a systematic review. J Laryngol Otol. 2012;126:8–14. 21867582 10.1017/S0022215111002039

[63] Marchioni D Rubini A Gazzini L. Complications in endoscopic ear surgery. Otol Neurotol. 2018;39:1012–1017. 30113561 10.1097/MAO.0000000000001933

[64] Ryan P Wuesthoff C Patel N. Getting started in endoscopic ear surgery. J Otol. 2020;15:6–16. 32110235 10.1016/j.joto.2018.10.002 PMC7033523

[65] McCallum R McColl J Iyer A. The effect of light intensity on image quality in endoscopic ear surgery. Clin Otolaryngol. 2018;43:1266–1272. 29768732 10.1111/coa.13139

[66] Fina M Chieffe D. Office-based otology procedures. Otolaryngol Clin North Am. 2019;52:497–507. 30905565 10.1016/j.otc.2019.02.004

